# The cAMP-PKA pathway regulates prey sensing and trap morphogenesis in the nematode-trapping fungus *Arthrobotrys oligospora*

**DOI:** 10.1093/g3journal/jkac217

**Published:** 2022-08-22

**Authors:** Sheng-An Chen, Hung-Che Lin, Yen-Ping Hsueh

**Affiliations:** Institute of Molecular Biology, Academia Sinica, Section 2, Nangang, Taipei 115, Taiwan; Institute of Molecular Biology, Academia Sinica, Section 2, Nangang, Taipei 115, Taiwan; Institute of Molecular Biology, Academia Sinica, Section 2, Nangang, Taipei 115, Taiwan

**Keywords:** nematode-trapping fungi, *Arthrobotrys oligospora*, cAMP signaling pathway, PKA, predator–prey interaction

## Abstract

Sensing environmental factors and responding swiftly to them is essential for all living organisms. For instance, predators must act rapidly once prey is sensed. Nematode-trapping fungi (NTF) are predators that use “traps” differentiated from vegetative hyphae to capture, kill, and consume nematodes. These traps undergo drastic and rapid morphological changes upon nematode induction. Multiple signaling hubs have been shown to regulate this remarkable process. Here, we demonstrate that the conserved cAMP-PKA signaling pathway exerts a crucial role in trap morphogenesis of the nematode-trapping fungi *Arthrobotrys oligospora*. A gene deletion mutant of the PKA catalytic subunit *TPK2* proved insensitive toward nematode presence. Moreover, we show that the G protein alpha subunit *GPA2* acts upstream of adenylate cyclase, with *GPA2* deletion resulting in substantially reduced trap formation, whereas exogenous provision of cAMP rescued the prey-sensing and trap morphogenesis defects of a *gpa2* mutant. Thus, we show that cAMP production triggered by G protein signaling and downstream PKA activity are vital for prey-sensing and trap development in *A. oligospora*, demonstrating that this highly conserved signaling pathway is critical for nematode-trapping fungi and nematode predator–prey interactions.

## Introduction

Detecting and responding to extracellular stimuli are crucial to the survival and reproduction of all life-forms. For example, during interspecies interactions, cues from the participating organisms activate conserved signaling cascades, such as the mitogen-activated protein kinase (MAPK) pathways and cyclic AMP (cAMP)-protein kinase A (PKA), which control key regulatory events (Lengeler *et al.* 2000; Mehrabi *et al.* 2009). In host–pathogen interactions, the pathogens often undergo cellular differentiation upon sensing specific host-derived signals, enabling them to effectively infect the host species. The virulence of diverse plant and animal pathogenic fungi is associated with drastic morphological switching, rendering them ideal systems for studying signaling transduction during host–pathogen interactions (Borges-Walmsley and Walmsley 2000). Roles for the cAMP-PKA and MAPK pathways in morphological differentiation and virulence have been well-established for several fungal pathogens, such as rice blast fungus *Magnaporthe oryzae* (Wilson and Talbot 2009), maize smut fungus *Ustilago maydis* (Brefort *et al.* 2009), and the human pathogen *Cryptococcus neoformans* (Idnurm *et al.* 2005).

Like pathogenic species, many fungal predators have evolved mechanisms to alter their morphologies in the pursuit of prey. Nematode-trapping fungi (NTF) are a group of nematophagous predators that form specialized mycelial structures known as “traps” to capture and kill nematodes as an additional source of nutrients (Nordbring-Hertz 2004; Vidal-Diez de Ulzurrun and Hsueh 2018). The formation of a functional adhesive network, or trap, consists of a series of hyphal fusion events. The initial trap cells grow as branching hyphae that are perpendicular to the vegetative hyphae. While elongating and curling, the trap initially subdivides into 3 cells that ultimately fuse with parental hyphae, resulting in a 3-cell loop capable of capturing nematodes (Nordbring-Hertz 2004). Trap morphogenesis represents a remarkable saprotrophic-to-predatory switch in the NTF lifestyle, with the process being highly analogous to appressorium formation in fungal plant pathogens. Recent studies have identified multiple MAPK pathways associated with trap development in the NTF *Arthrobotrys oligospora* (Zhen *et al.* 2018; Kuo *et al.* 2020; Chen *et al.* 2021; Xie *et al.* 2021). Highly conserved MAPKs responsible for fungal cell wall integrity and the pheromone response pathway both play vital roles in *A. oligospora* trap differentiation. Disruption of the Slt2 MAPK severely disrupted trap morphology and reduced the sensitivity of *A. oligospora* toward nematodes (Zhen *et al.* 2018; Chen *et al.* 2021), whereas deletion of the Fus3 MAPK abolished trap morphogenesis (Chen *et al.* 2021). *Arthrobotrys oligospora hog1* MAPK mutants, in which the hyperosmotic pathway is impaired, display inhibited trap formation and diminished predatory efficiency, although overall trap structure is not affected (Kuo *et al.* 2020).

The cAMP-dependent protein kinase A (cAMP-PKA) signaling pathway, which is highly conserved and essential for cellular differentiation throughout the fungal kingdom, represents another signaling hub likely important in triggering trap differentiation in NTF (Bahn *et al.* 2007; Li *et al.* 2007; Xie *et al.* 2019; Yang *et al.* 2021; Bai *et al.* 2022). The PKA of budding yeast *Saccharomyces cerevisiae* is a heterotetrameric complex comprised of 2 regulatory subunits (Bcy1p) that bind to 2 catalytic subunits (Toda, Cameron, Sass, Zoller, Scott *et al.* 1987; Toda, Cameron, Sass, Zoller, Wigler 1987; Kuret *et al.* 1988). G-protein alpha subunits (Gα) act as pivotal regulators upstream of the cAMP-PKA pathway (Li *et al.* 2007). Gpa2, a Gα subunit of *S. cerevisiae*, activates the adenylate cyclase Cyr1 to enhance cAMP production and subsequently upregulate PKA signaling (Colombo *et al.* 1998; Kays *et al.* 2000). The PKA heterotetrameric complex is inactive in the absence of cAMP. Upon production of cAMP by adenylate cyclase, cAMP-binding by the 2 Bcy1p regulatory subunits causes their dissociation from the complex, thereby releasing the catalytic subunits as active monomers to regulate downstream targets via phosphorylation (Toda, Cameron, Sass, Zoller, Scott *et al.* 1987; Toda, Cameron, Sass, Zoller, Wigler 1987; Borges-Walmsley and Walmsley 2000). PKA controls *S. cerevisiae* cell shape in response to nutrients, with nitrogen starvation-induced intracellular cAMP levels activating PKA and filamentous growth (Pan and Heitman 1999; Borges-Walmsley and Walmsley 2000). Moreover, PKA signaling is crucial to the virulence of many fungal pathogens. For instance, the addition of exogenous cAMP to *M. oryzae* was shown to directly induce differentiation of appressoria (Lee and Dean 1993), specialized cells for infecting plant hosts. Molecular characterizations of components within the cAMP-PKA pathway have also demonstrated the importance of PKA signal transduction to host recognition and appressorium formation in *M. oryzae* (Mitchell and Dean 1995; Ramanujam and Naqvi 2010). The contribution of cAMP-PKA signaling to the yeast-hyphal transition and pathogenesis has been well described for *U. maydis*, in which PKA activity-deficient mutants display filamentous growth; a state in which conjugation tubes cannot form between mating-compatible cells to produce the pathogenic dikaryon (Dürrenberger *et al.* 1998; Lanver *et al.* 2017). In the human pathogen *C. neoformans*, cAMP signaling links nutrient sensing to downstream virulence factors, such as capsule formation and melanization (D'Souza *et al.* 2001).

Here, we demonstrate that the cAMP-PKA pathway of the NTF *A. oligospora* is crucial for trap morphogenesis and virulence against nematodes. We demonstrate that both GPA2, the G protein α subunit that acts upstream of the cAMP-PKA signaling pathway, and the catalytic PKA subunit *TPK2* are required for nematode-sensing and trap-development. Our study provides the first genetic proof demonstrating roles for the G protein-cAMP-PKA pathway in prey sensing and trap development in NTF.

## Materials and methods

### Strains and culture conditions

The *A. oligospora* strains used in this study are listed in Supplementary Table 1. Fungal strains were maintained routinely on potato dextrose agar (PDA; Difco). For trap induction, fungal cultures were maintained on low-nutrient medium agar (LNM: 2% agar, 1.66 mM MgSO_4_, 5.4 µM ZnSO_4_, 2.6 µM MnSO_4_, 18.5 µM FeCl_3_, 13.4 mM KCl, 0.34 µM biotin, and 0.75 µM thiamin). Liquid culturing of fungi to acquire protoplasts was conducted in potato dextrose broth (PDB; Difco). *Caenorhabditis elegans* N2 strain was maintained on standard NGM medium with *Escherichia coli* (OP50) as the food source. The additional details regarding laboratory culture and trap assay of *A. oligospora* are as described previously (Lin and Hsueh 2021).

### Identification of PKA catalytic subunits in *A. oligospora*

The catalytic subunits of cAMP-dependent PKA in *A. oligospora* were identified by using protein sequences of known orthologs of the model fungal species *S. cerevisiae*, *Neurospora crassa*, *M. oryzae*, and *Aspergillus nidulans* to search the *A. oligospora* genome (NCBI, accession number: SOZJ00000000) using Blast2GO 5 PRO (Conesa *et al.* 2005), which revealed 2 predicted catalytic subunits, i.e. *TPK1* (EYR41_008633) and *TPK2* (EYR41_006022). The positions of functional domains in *TPK1* and *TPK2* were located by searching the protein sequences in the PROSITE database using ScanProsite (de Castro *et al.* 2006; Sigrist *et al.* 2013).

### Phylogenetic analysis of *A. oligospora* PKA catalytic subunits and Gα subunits

The phylogenetic relationships of the PKA catalytic subunits and Gα subunits from *A. oligospora* and model fungi were assessed by aligning full-length protein sequences in ClustalW, and then creating a neighbor-joining phylogenetic tree of the respective sequences using Mega 7 (Kumar *et al.* 2016), with 3,000 bootstrap replicates to evaluate clade support.

### Generation of *A. oligospora* gene deletion mutants

Targeted gene deletions were carried out in a *ku70* mutant strain of *A. oligospora* TWF154, as its inherent NHEJ deficiency boosts the genomic recombination rate (Kuo *et al.* 2020). In brief, 1.5 kilobases (kb) of the 5′ and 3′ regions flanking the open reading frame (ORF) of the target gene were amplified and fused to a clonNAT resistance cassette (Chee and Haase 2012) to generate the knockout constructs. The constructs were then introduced into protoplasts of the *ku70* background (*A. oligospora* TWF1697) via PEG-mediated transformation. To acquire fresh protoplasts, 5 × 10^6^ conidia of *A. oligospora* TWF1697 were inoculated in 100 ml of PDB for 48 h at 25°C and 200 rpm. Blended mycelia were mixed with 50 mg/ml VinoTastePro lytic enzyme in MN buffer (0.3 M MgSO_4_, 0.3 M NaCl) and incubated for 5 h at 25°C and 200 rpm. The protoplasts were collected by filtering the mixture through 3 layers of miracloth and then washing with STC buffer (1.2 M sorbitol, 50 mM CaCl_2_, 10 mM Tris-HCl pH 7.5). Transformations to acquire knockout mutants were performed by mixing protoplasts with 5 µg of construct DNA, incubating on ice for 30 min, and then adding 5 volumes of PTC buffer [40% PEG 4000 (w/v), 10 mM Tris-HCl pH 7.5, 50 mM CaCl_2_].

Successful knockouts of target genes were first confirmed by polymerase chain reaction (PCR), and then validated by Southern blotting to establish the presence of the ectopically integrated drug cassette (Supplementary Fig. 1). For gene complementation of deletion strains, wild-type gene copies (1.5–2 kb upstream and 0.5 kb downstream of the ORF) were fused to a G418 resistance cassette (Youssar *et al.* 2019) by PCR and transformed into the protoplasts of targeted mutant strains.

### Quantification of *A. oligospora* trap morphogenesis in response to *C. elegans*

Evaluations of the number of traps formed by *A. oligospora* in response to *C. elegans* were carried out as described previously (Chen *et al.* 2021). In brief, 30 *C. elegans* nematodes at the L4 developmental stage were added to *A. oligospora* cultures that had been grown on an LNM plate (2.5 cm) for 2 days at 25°C. The nematodes were removed after 6 h and, 24 h after nematode induction, 3 images were taken randomly within 0.5 cm of the plate edge under a dissecting microscope at 40× magnification. Traps in each of the 3 images were quantified using “Fungal Feature Tracker” (Vidal-Diez de Ulzurrun *et al.* 2019) and summed as a representative score of trap formation in each plate.

### Survival rate of *C. elegans* upon exposure to *A. oligospora*

The evaluations of *C. elegans* survival rate upon exposure to *A. oligospora* were carried out as described in Lin and Hsueh (2021), but with a slight modification. In brief, the fungal strain was inoculated onto a 2.5-cm LNM plate for 2 days at 25°C. Then, 80 young adult *C. elegans* nematodes were added onto the fungal culture. Numbers of worms caught in fungal traps were counted manually every 2 h for 24 h using a stereomicroscope.

## Results

### Disruption of PKA catalytic subunit Tpk2 abolishes trap morphogenesis in *A. oligospora*

**Fig. 1.**
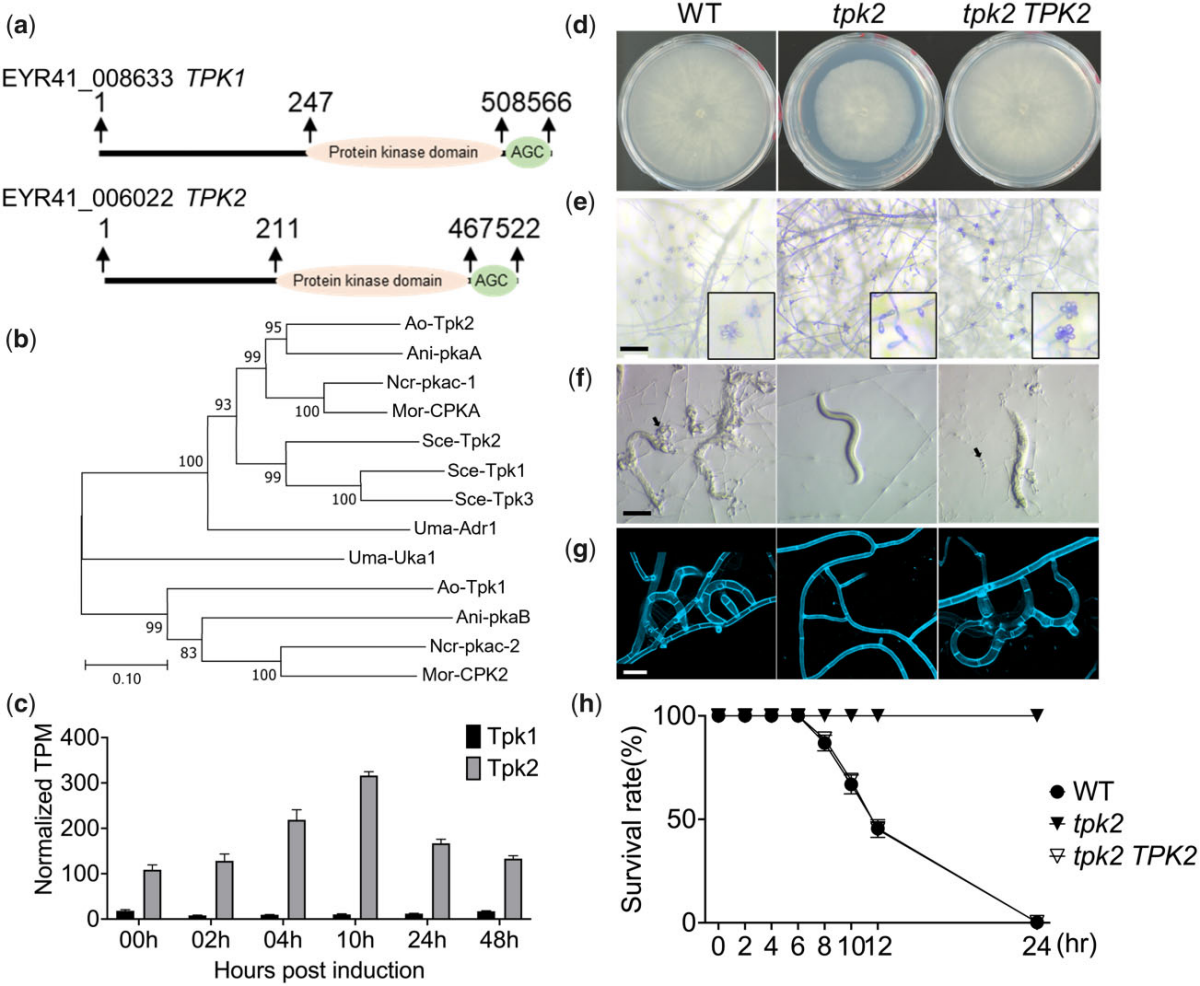
Disruption of the catalytic subunit Tpk2 of PKA results in defective trap morphogenesis in *A. oligospora*. a) Domain description of PKA catalytic subunits *TPK1* and *TPK2* in *A. oligospora*. Positions of the protein kinase domain (PROSITE entry #PS50011) and AGC-kinase C-terminal domain (AGC; PROSITE entry #PS51285) in the Tpk1 and Tpk2 protein sequences were determined using ScanProsite. b) A neighbor-joining phylogenetic tree of PKA catalytic subunit protein sequences from *A. oligospora* and orthologs from model fungi. Ani: *A. nidulans*. Ao: *A. oligospora*. Mor: *M. oryzae.* Ncr: *N. crassa.* Sce: *S. cerevisiae.* Uma: *U. maydis*. c) Normalized transcripts per million (TPM) of *TPK1* and *TPK2* in a time-course transcriptomic analysis in response to *C. elegans* nematodes. d) Colony morphologies of wild-type (*ku70*), *tpk2* mutant, and the *tpk2 TPK2*-complemented strain after 4 days at 25°C on PDA plates (5 cm diameter). e) Conidiation by mutant and complemented strains was recorded after culturing for 4 days on PDA plates (scale bar, 200 μm). f) Trap induction of mutant and complemented strains. Trap formation was induced by adding 30 *C. elegans* nematodes to fungal cultures on LNM plates (2.5 cm), and images were taken 24 h after induction (scale bar, 200 μm). g) Close-up images of *A. oligospora* traps after 24 h of continuous nematode exposure. Vegetative hyphae and traps of *A. oligospora* were stained with SR2200, which specifically binds to fungal cell walls (scale bar, 20 μm). h) Nematode survival assay of mutant and complemented strains. Survival rate of nematodes for each timepoint was calculated by dividing the number of living nematodes by the total number of nematodes at timepoint zero.

Most fungal pathogens have 2 genes encoding the catalytic subunits of PKA, with only one having a major role in the infection process (Mehrabi *et al.* 2009). For example, the CPKA catalytic subunit of *M. oryzae* is essential for pathogenicity, but impairment of the second subunit CPK2 elicits no obvious defects in host infection (Thines *et al.* 2000). The *A. oligospora* genome encodes 2 predicted catalytic PKA subunits, i.e. *TPK1* (EYR41_008633) and *TPK2* (EYR41_006022), with both genes hosting catalytic serine/threonine protein kinase domains alongside AGC-kinase C-terminal domains that harbor conserved phosphorylation sites for regulating PKA function (Fig. 1a). Our phylogenetic analysis of the catalytic PKA subunits from model fungi revealed that *TPK2* of *A. oligospora* occurs in the same clade as *CPKA* of *M. oryzae* and *ADR1* of *U. maydis*, representing the primary catalytic subunits responsible for plant infection, respectively (Fig. 1b). In addition, we detected that *TPK2* was upregulated in the *A. oligospora* transcriptome in response to nematode presence, whereas there was no change in *TPK1* expression (Fig. 1c). Accordingly, we hypothesized that *TPK2* may play a major role in prey sensing and trap morphogenesis by *A. oligospora*.

To test this hypothesis, we acquired independent *TPK2* deletion mutants in the *ku70* background (TWF1697) in which the homologous recombination rate is higher, facilitating targeted gene deletion (Kuo *et al.* 2020), and examined the resulting phenotypes under a variety of conditions. First, we assessed the growth of the *tpk2* mutant. On rich medium (PDA), the *tpk2* mutant grew slightly slower than wild type and exhibited an obvious conidiation defect, with only 1 or 2 conidia on the conidiophore, contrasting with the rosette cluster observed in the wild type (Fig. 1, d and e). Both the hyphal growth and conidiation defects could be complemented by introducing a wild-type copy of *TPK2*, demonstrating that the phenotypes were attributable to *tpk2* deletion. Next, we examined the prey-sensing ability and trap morphogenesis of the *tpk2* mutant. When we exposed wild-type *A. oligospora* to *C. elegans*, numerous adhesive traps developed and they efficiently captured the nematodes (Fig. 1f). However, in the *tpk2* mutant, hyphae remained undifferentiated in the presence of nematodes, demonstrating that trap morphogenesis has been completely abolished in the *tpk2* mutant (Fig. 1, f and g), meaning these mutants were unable to capture nematodes. When we measured the survival rate of *C. elegans* 24 h following exposure to *A. oligospora*, 100% of the animals survived in the presence of the *tpk2* mutant, in contrast to 0% survival observed in coculture with the wild-type fungus (Fig. 1h). The defects in trap development and prey capture were rescued in a wild-type *TPK2*-complemented strain. Together, these results demonstrate that the Tpk2 catalytic subunit of PKA regulates hyphal growth, conidiation, and trap development in the NTF *A. oligospora*. Without *TPK2*, *A. oligospora* could not initiate trap morphogenesis to catch the nematode prey.

### The G protein α subunit Gpa2 is required for prey-sensing and trap morphogenesis

**Fig. 2.**
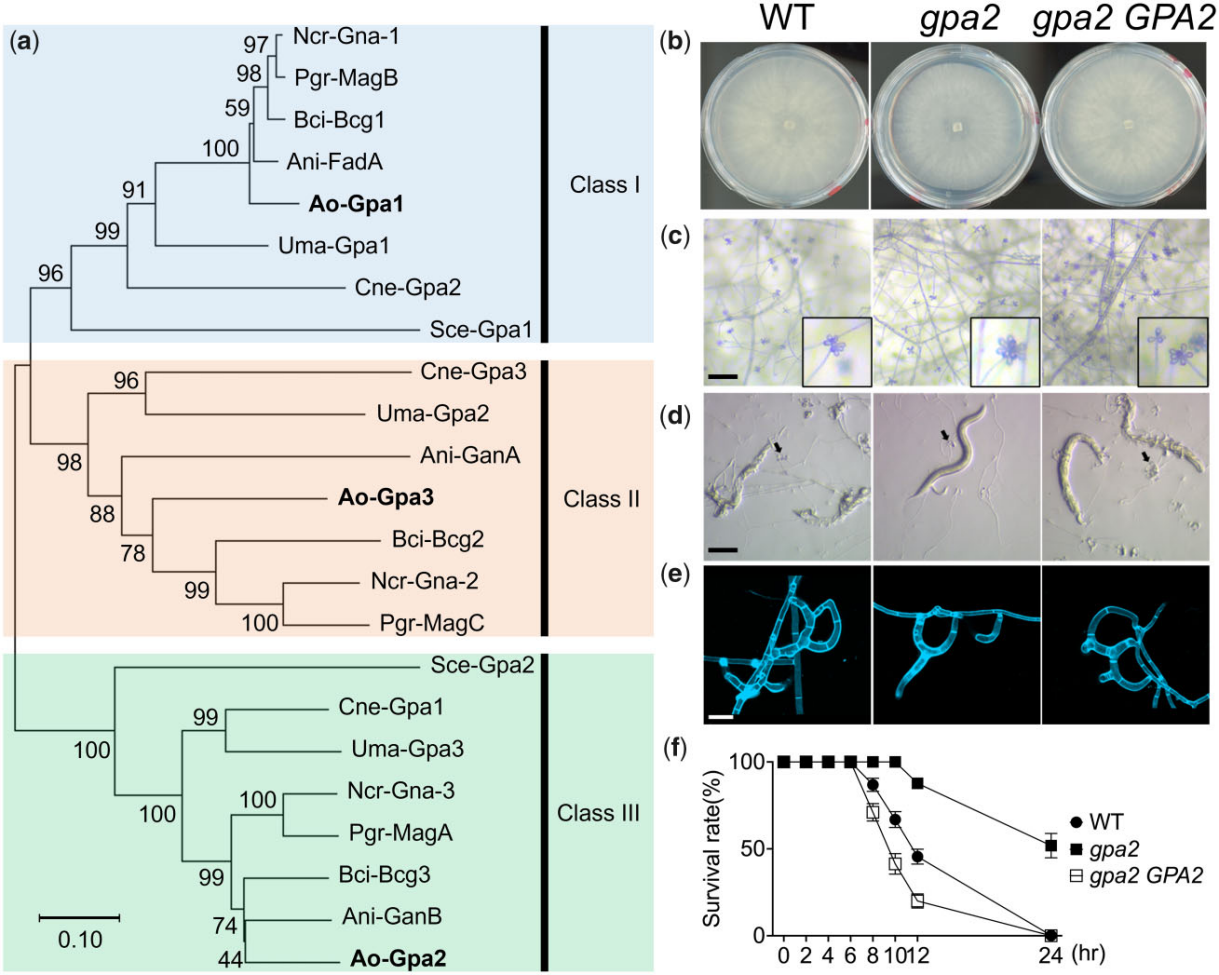
G protein alpha subunit Gpa2 is vital for formation of a complete adhesive network. a) A neighbor-joining phylogenetic tree of Gα subunit protein sequences from *A. oligospora* and orthologs from model fungi. Ani: *A. nidulans*. Ao: *A. oligospora*. Bci: *Botrytis cinerea*. Cne: *C. neoformans*. Ncr: *N. crassa.* Pgr: *Pyricularia grisea.* Sce: *S. cerevisiae.* Uma: *U. maydis*. b) Colony morphologies of wild-type (*ku70*), *gpa2* mutant, and *gpa2 GPA2* complemented strains after 4 days on PDA plates. c) Conidiation by mutant and complemented strains after 4 days on PDA plates (scale bar, 200 μm). d) Trap induction of *gpa2* mutant and complemented strains. Images were taken 24 h after induction (scale bar, 200 μm). e) Close-up images of traps of *gpa2* mutant and complemented strains after 24 h of continuous nematode exposure. Vegetative hyphae and traps were stained with SR2200 (scale bar, 20 μm). f) Nematode survival assay of *gpa2* mutant and complemented strains. Survival rate of nematodes for each timepoint was calculated by dividing the number of living nematodes by the total number of nematodes at timepoint zero.

G protein α (Gα) subunits are known to regulate intracellular cAMP levels and downstream PKA activity in fungi (Li *et al.* 2007; Mehrabi *et al.* 2009). Three Gα subunits are encoded in the *A. oligospora* genome. To identify which Gα subunit is most likely to operate upstream of PKA in NTF, we built a neighbor-joining phylogenetic tree based on the protein sequences of known Gα subunits from various model fungi and *A. oligospora* (Fig. 2a). From our phylogenetic analysis, it is clear that *A. oligospora GPA2* (EYR41_010456) displays highest sequence similarity to group III Gα subunits, which function in the nutrient-sensing pathway and regulate cAMP production in the budding yeast *S. cerevisiae* and in other fungi (Li *et al.* 2007). To determine if *A. oligospora GPA2* acts upstream of the cAMP-PKA pathway and plays a major role in transducing nematode signals in this NTF, we conducted targeted gene deletion of *GPA2* in *A. oligospora.* As shown in Fig. 2b, deletion of *GPA2* resulted in a very mild growth defect on nutrient-rich PDA medium compared to wild-type *A. oligospora*, but conidiation was not affected (Fig. 2, b and c). Next, we examined if *GPA2* is involved in prey-sensing and trap morphogenesis by exposing the *gpa2* mutant to *C. elegans*. In contrast to wild-type *A. oligospora* that developed numerous traps within 24 h of exposure to *C. elegans* and efficiently caught all the nematode prey, the *gpa2* mutant displayed a dramatic reduction in the number of traps it developed and it was unable to catch *C. elegans* efficiently (Fig. 2, d and e). Moreover, unlike the mature traps composed of 2 or 3 adhesive loops developed by wild-type cultures, we observed trap morphology was very much less complex in the few traps developed by the *gpa2* mutant, consisting of only a single or half loop after 24 h of nematode exposure (Fig. 2e). Only a small proportion of these partial traps eventually developed into mature adhesive networks after 48 h, suggesting trap development was halted in the *gpa2* mutant. To examine the functionality of the “stunted” traps formed by the *gpa2* mutant, we tested *C. elegans* survival on cultures of wild-type and *gpa2* lines. In contrast to wild-type and the *GPA2*-complemented mutant strains that captured and killed all nematodes within 24 h, ∼50% of nematodes remained alive on the *gpa2* mutant (Fig. 2f). These results demonstrate that Gpa2 plays a key role in prey-sensing, trap development, and prey capture in *A. oligospora*.

### *GPA2* acts upstream of the cAMP-PKA pathway

**Fig. 3.**
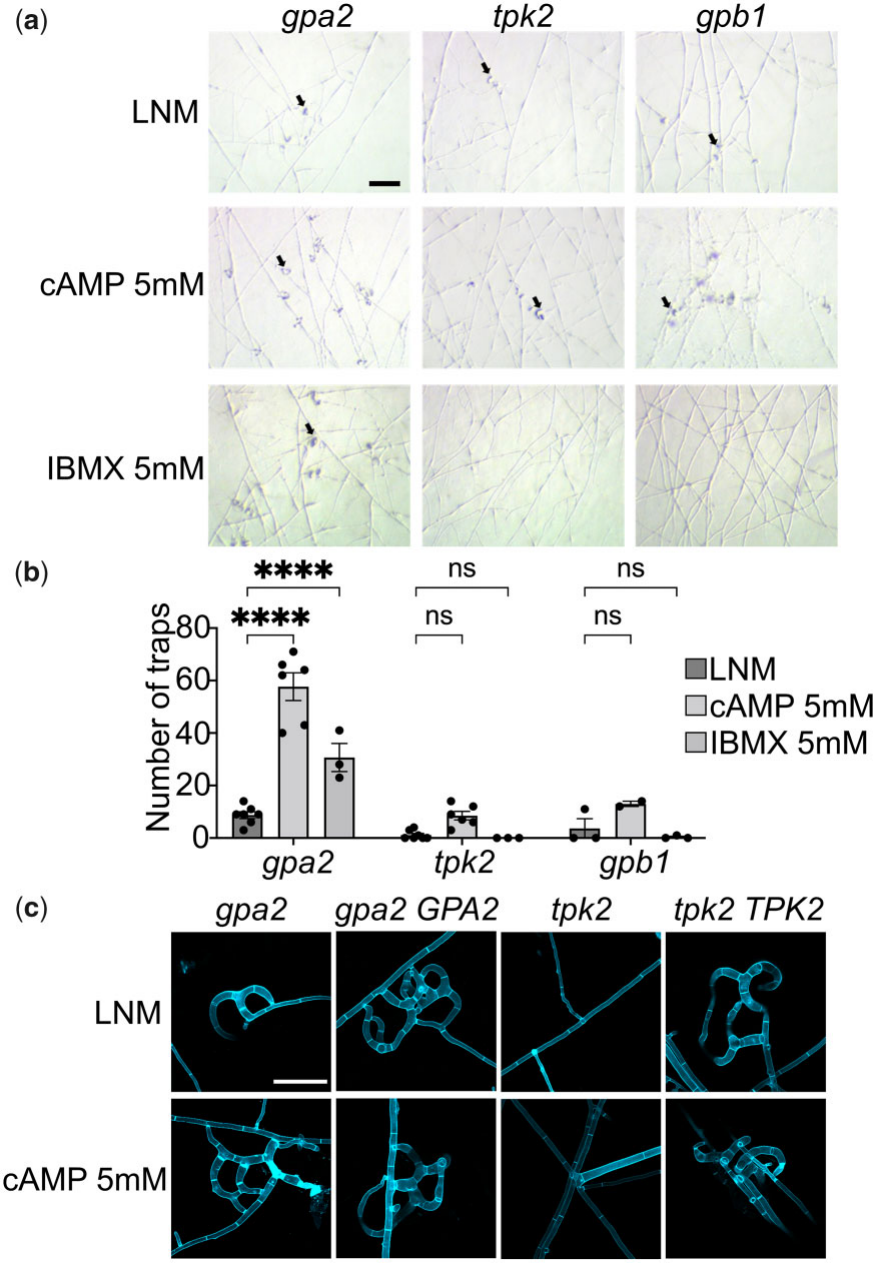
Exogenous cAMP restores trap formation in the *gpa2* mutant line. a) Trap induction by *gpa2*, *tpk2*, and *gpb1* mutants on LNM and LNM supplemented with 5 mM cAMP or 5 mM IBMX (a nonspecific inhibitor of cAMP phosophodiesterases; 2.5 cm plates). *Caenorhabditis elegans* (*n* = 30) nematodes were added to each plate for 6 h and then washed away. Images were taken 24 h after nematode induction (scale bar, 200 μm). b) Trap quantification of the mutant strains on LNM and LNM supplemented with 5 mM cAMP or 5 mM IBMX (mean ± SEM). c) Close-up images of traps after 24 h of continuous nematode exposure. Vegetative hyphae and traps were stained with SR2200 (scale bar, 40 μm).

To determine if Gpa2 acts directly upstream of the cAMP-PKA pathway, we assessed if exogenous cAMP could rescue the defective trap development of the *gpa2* mutant. When we supplied 5 mM exogenous cAMP into the medium, trap development by the *gpa2* mutant was restored. Furthermore, addition of IBMX (3-isobutyl-1-methylxanthine), a nonselective phosphodiesterase inhibitor that raises intracellular cAMP levels, also significantly increased the number of traps developed in response to *C. elegans* by the *gpa2* mutant (Fig. 3, a–c). In addition, we also examined the response of *gpb1* mutant to exogenous cAMP and IBMX. *GPB1* encodes for the only G protein β subunit in *A. oligospora*, and the deletion mutant *gpb1* abolished trap formation (Yang *et al.* 2020). Previous evidence suggested Gpb1 acts upstream of the pheromone-response MAPK pathway in *A. oligospora* (Chen *et al.* 2021), but the link between Gpb1 and cAMP-PKA remains uncharacterized. We found that exogenous cAMP or IBMX did not enhance trap development in *gpb1* or *tpk2* mutants (Fig. 3, a and b), demonstrating that *GPB1* does not act upstream of the cAMP-PKA pathway and that without *TPK2*, increasing the intracellular cAMP level does not activate this pathway. Together, these results support the model that in the presence of *C. elegans*, Gpa2 activates the downstream cAMP-PKA pathway to initiate trap development in *A. oligospora.*

## Discussion

Morphological switching from vegetative hyphae to trap cells is a distinctive feature of NTF. In *A. oligospora*, trap development represents a series of hyphal fusion events, resulting in a 3-cell adhesive network that differs significantly from the rest of the mycelium in terms of structure and function. Not only do the trap cells host unique cytosolic organelles, termed “dense bodies,” absent from vegetative hyphae, they also feature an extracellular adhesive layer that is thought to mediate adhesion of nematodes to the traps (Veenhuis *et al.* 1985; Rosén *et al.* 1992). During the early stages of trap formation in *A. oligospora*, genes related to translation, amino acid metabolism, carbohydrate metabolism, and cell wall/membrane biogenesis were reported to be significantly upregulated (Yang *et al.* 2011), implying that multiple biological processes are involved in the morphological switch. However, the signaling transduction pathways governing trap morphogenesis remain largely uncharacterized. Recent studies have shown that the highly conserved Slt2 MAPK of the cell wall integrity pathway (Zhen *et al.* 2018) and the Fus3 MAPK of the pheromone response pathway (Chen *et al.* 2021) both play vital roles in *A. oligospora* trap differentiation, with Fus3 signaling being essential to induce trap morphogenesis. Moreover, lack of the SipC component of the STRIPAK (striatin-interacting phosphatases and kinases) complex in another species of NTF (*Duddingtonia flagrans*) that forms 3-dimensional traps results in incomplete loop formation and column-like trap structures (Wernet *et al.* 2022). A recent report linked intracellular cAMP signaling to prey sensing in *A. oligospora*, which demonstrated that deletion mutants of 2 cAMP phosphodiesterases, PdeH and Pdel, resulted in increased cAMP levels during vegetative growth and trap morphogenesis. Furthermore, a *pdeH* mutant displayed a significant growth defect and also lacked the capability to differentiate traps (Ma *et al.* 2022). However, the catalytic and regulatory subunits within the cAMP-dependent PKA complex have not yet been characterized for any species of NTF.

Here, we have demonstrated that cAMP-PKA plays a significant role in prey sensing and trap formation by *A. oligospora*. A genetic mutant line for the Tpk2 catalytic subunit could not form traps, with the vegetative hyphae of this mutant remaining undifferentiated upon exposure to *C. elegans*. Only rarely did we observe *tpk2* mutants initiating trap formation, but the slightly curved hyphae never progressed to developing into adhesive loops. Furthermore, *tpk2* mutant also exhibited mild conidiation defects, suggesting that *TPK2* is involved in multiple developmental pathways.

Most plant pathogenic fungi possess 2 genes encoding the PKA catalytic subunits, although generally only one plays a primary role in plant host infection and the other appears to have no direct impact on pathogenesis; the catalytic subunits *CPKA* in *M. oryzae* and *ADR1* of *U. maydis* are good examples (Mehrabi *et al.* 2009). As described for these latter pathogenic fungi, we found that disrupting just one of its 2 catalytic subunits abolished *A. oligospora* predatory ability. The *tpk2* mutant also exhibited slower growth rates on both rich and nutrient-deficient media, as well as a clear reduction in conidiation. Based on studies of cAMP-PKA pathways in fungal plant pathogens, the 2 PKA catalytic subunits may have overlapping or distinct roles during infection and vegetative growth (Mehrabi *et al.* 2009). For instance, deletion of the primary catalytic subunit responsible for virulence in *M. oryzae*, *CPKA*, elicits no obvious defect in vegetative growth as the functions of the second catalytic subunit (*CPK2*) overlap in this respect, but *cpkA*Δ*cpk2*Δ double mutants exhibit significantly reduced growth and conidiation rates compared to the single deletion mutants (Selvaraj *et al.* 2017). The roles of the other catalytic subunit Tpk1 encoded in the *A. oligospora* genome (EYR41_008633) and of the regulatory PKA subunit Bcy1 (EYR41_011649) remain to be elucidated. We endeavored to acquire a targeted adenylate cyclase *CYR1* (EYR41_002049) deletion mutant, but failed despite multiple rounds of transformation.

Heterotrimeric G proteins exert universal roles as signaling proteins in eukaryotes. The heterotrimer is composed of alpha (Gα), beta (Gβ), and gamma (Gγ) subunits that are associated with the G-protein-coupled receptors (GPCRs) on plasma membranes. Upon ligands binding to the GPCRs, GDP-GTP exchange on the Gα subunit results in dissociation of the Gα•Gβγ dimer. Accumulating evidence supports that G-proteins positively regulate the cAMP-PKA pathway in filamentous fungi. Mutations of Group I or III Gα, Gβ, or Gγ genes have been shown to alter intracellular cAMP levels and manifest as defective phenotypes in fungi (Li *et al.* 2007; Mehrabi *et al.* 2009). We generated a genetic mutant of Gpa2, representing the Gα subunit in *A. oligospora* displaying the highest sequence similarity to group III Gα proteins in diverse filamentous fungi. We found that disruption of Gpa2 signaling significantly impaired the ability of *A. oligospora* to develop traps, both in terms of quantity and morphology. The addition of exogenous cAMP rescued the phenotypic defects of the *gpa2* mutant, evidencing that Gpa2 of *A. oligospora* mediates signaling to the cAMP-PKA pathway, which subsequently activates the downstream targets required for proper trap morphogenesis. Intriguingly, exogenous cAMP did not restore the defective trap development exhibited by a *gpb1* mutant (Chen *et al.* 2021), implying that Gpb1 regulates trap formation via pathways independent of cAMP-PKA, perhaps the pheromone-response MAPK cascade. Furthermore, *gpa2* mutant exhibited slower growth than wild-type, but this phenotype was less pronounced than that of the *tpk2* mutant; conidiation was not affected in *gpa2*, suggesting that *TPK2*’s role in conidiation might be a result of more complex crosstalk between different signaling pathways. Apart from Gpa2 and Gpb1, the remaining 2 Gα and 1 Gγ subunits in *A. oligospora* have yet to be functionally characterized. Furthermore, the GPCRs acting upstream of Gpa2 and their nematode-derived ligands warrant investigation.

**Fig. 4.**
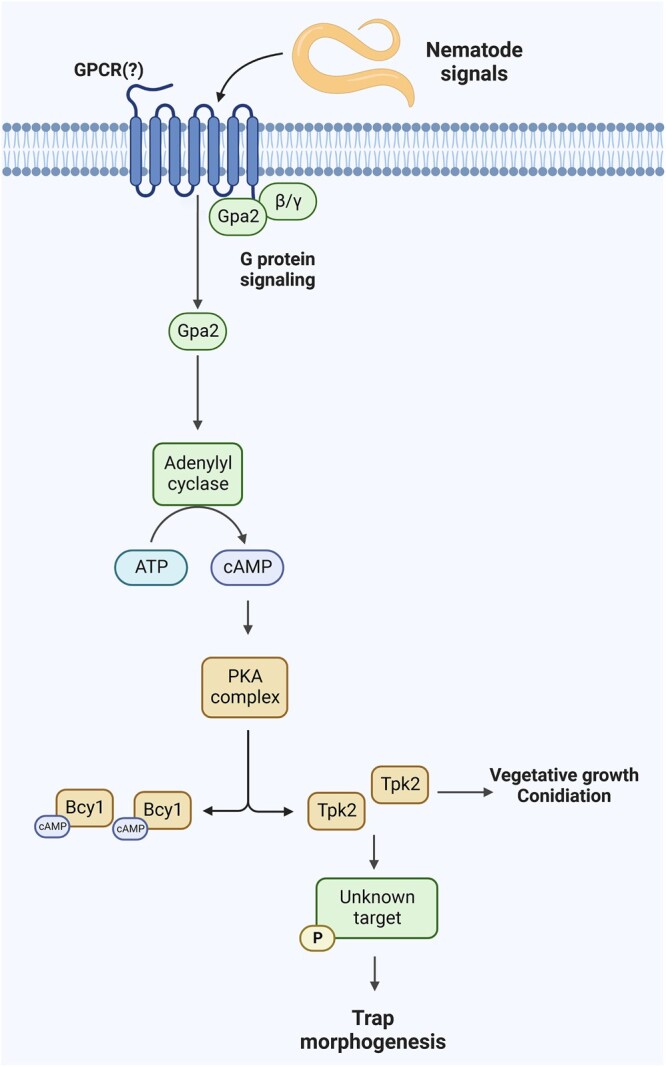
Hypothetical model of cAMP-PKA signaling during prey sensing and trap development in *A. oligospora*. Nematode-derived signals activate unidentified GPCRs of *A. oligospora*, leading to dissociation of the heterotrimeric G-protein complex and release of the Gα Gpa2. Gpa2 then activates the downstream adenylate cyclase Cyr1, leading to an increase in cAMP levels. cAMP binds to regulatory Bcy1 subunits of PKA to activate its Tpk2 catalytic subunits that phosphorylate the downstream substrates required for trap morphogenesis.

Our study has characterized the key components of the cAMP-PKA pathway in NTF, and we have demonstrated that PKA acts as a crucial regulator of trap morphogenesis in *A. oligospora*. A hypothetical model of the Gpa2-cAMP-PKA pathway during prey sensing in NTF is illustrated in Fig. 4. Our future investigations will focus on discovering downstream targets of PKA and identifying GPCRs that interact with the G-proteins. Elucidating the signaling transductions responsible for trap differentiation will not only enhance our understanding of how NTF sense and respond to their nematode prey, but will also provide valuable insights into the origin and evolution of this remarkable interkingdom interaction.

## Supplementary Material

jkac217_Supplemental_Material

jkac217_Table_S1_TableS2

## Data Availability

Strains and plasmids are available upon request. The authors affirm that all data necessary for confirming the conclusions of the article are present within the article, figures, and tables. Supplemental material is available at G3 online.
