# Supplementary material for: The cAMP-PKA pathway regulates prey sensing and trap morphogenesis in the nematode-trapping fungus Arthrobotrys oligospora
Source: G3 (Bethesda). 2022 Aug 22;12(10):jkac217. doi: 10.1093/g3journal/jkac217 (PMC9526039; doi:10.1093/g3journal/jkac217)
Supplement: jkac217_Supplemental_Material [file jkac217_supplemental_material.docx]

**Figure S1. Southern blot confirmation of the *A. oligospora* *tpk2* and *gpa2* deletion mutants.**

Probe design and Southern blots of the (A) *tpk2* and (B) *gpa2* deletion mutants used in this study. Multiple deletion mutants were acquired for *TPK2*, and the one highlighted in red (TWF3443) was further assayed.

**Table S1. Strains used in this study.**

**Table S2. Class type, gene name, and NCBI accession numbers for the Gα subunits used in the phylogenetic analysis from model fungi and *A. oligospora*.**
